# Poor-Law Infirmaries and Union Hospitals

**Published:** 1914-10-31

**Authors:** Henry Burdett


### REPORTS ON
### Hospitals of the United Kingdom.

By SIR HENRY BURDETT, K.C.B., K.C.V.O.

**SERIES III.**

### POOR-LAW INFIRMARIES AND UNION HOSPITALS.
### AN INQUIRY AND SCHEME SUGGESTED.

We have given full reports on the present condition and work of some of the best and some of the worst Poor-Law infirmaries, the latter being institutions of an old type, which must in a few years entirely disappear. At its best the up-to-date, modern Poor-Law infirmary, with its medical superintendent in responsible charge of all its departments, having the assistance and co-operation of one or two able medical colleagues, according to the number of beds it contains, and supported by the helpful co-operation of a trained and experienced nurse as lady superintendent or matron, where the architect has realised that the Poor-Law infirmary of to-day ought in fact to be so constructed as to be in the best sense a modern, up-to-date State hospital, can compare with any medical institution of its type and hold its own. In the past even better-designed Poor-Law infirmaries for the most part have suffered from (1) the absence of an adequate supply of trained nurses, from (2) the want of an operation unit well thought out and complete so as to fulfil to the full the requirements of modern teaching and experience, and (3) from the non-appointment of one or more surgeons of repute to enable every surgical case, however difficult and important, to be treated promptly in the ablest possible manner. Happily, in the last few years the Guardians in various places, stirred up by the zealous and self-denying efforts of capable medical superintendents, have adopted the latter's suggestions, and agreed to appoint outside surgeons and to provide a properly equipped operation unit.

### Guardians and Superintendents.

There yet remains, even in the best Poor-Law infirmary, an omission to recognise that you cannot properly treat acute disease and serious operation cases with justice to the patients, where the nursing staff is so small as to represent probably one nurse for every nine or ten patients. Before any Poor-Law infirmary can be properly converted into a State hospital it must be supplied with an adequate number of capable nurses of the best type. In saying this, we wish to emphasise that the omissions that we have indicated are really, so far as cost and change are concerned, relatively small matters, which a capable medical superintendent, who is given the necessary authority, can put right quickly and completely. It is important at the present time, in our judgment, to emphasise these points, seeing that the waiting-lists at the larger general hospitals up and down the country are so heavy, and that the number of urgent cases upon them requiring in-patient treatment are increasing to such an extent that it is essential that more hospital beds and more surgical and nursing skill shall be provided in addition to that which at present is available in the voluntary hospitals. Further, we are within little more than a year of the date when the actuaries will publish their first valuation exhibiting the results of the working of the National Insurance Act. We have little doubt that the actuaries' report will show many things which will somewhat startle the public and possibly hurt Friendly Societies, or some of them, grievously. In such an event the demand for adequate in-patient treatment will extend far beyond anything the hospitals have yet had to face, and it is only just to the sick, to the hospitals, and, indeed, to those whose credit is so closely mixed up with the success of National Insurance, that the probable issues to be faced shall be ascertained and carefully considered by the Insurance Commissioners and by the Government with the Treasury, upon whom the credit or discredit of success or failure will properly fall.

### From Infirmary to State Hospital.

As we have insisted on many previous occasions, the best of the Poor-Law infirmaries can readily and at small cost be put into a condition to enable them to efficiently undertake the best work of a modern, up-to-date hospital. It is well to remember that some thousands of beds can be made available by utilising the Poor-Law infirmaries and converting them into State hospitals—State hospitals, that is, in the sense that all pauper taint is effectually removed, and that it is made possible to convert some of their pavilions into paying wings, to which the patients can be admitted by payment of (*a*) the whole cost of their maintenance, or (*b*) the whole cost of their maintenance and medical treatment, or (*c*) those patients who desire to pay something according to their means, though it may not be the entire cost of even maintenance alone. Everywhere we find the strongest wish expressed amongst the working classes that they shall be free from all obligations to generous donors, and, in the alternative, from the taint of accepting rate support which traditional habit has made the English people of all classes regard as pauper relief. Consequently it is essential to bear these facts in mind, and at the same time to protect the national revenue to the fullest possible extent by falling in with the wishes of the population to maintain their independence in the day of illness, while seeking admission to a paying ward where they can satisfy their commendable wish to preserve their independence by contributing what they can towards the cost of their treatment and maintenance. We look forward to the time when there will be, in connection with every voluntary hospital, paying pavilions, where patients who prefer to adopt that course can, as in the United States, obtain hospital care and treatment by payment varying from the actual cost of the simplest form of accommodation to that of the highest and most expensive grade. Such pavilions, though administered by the voluntary hospital authorities, will have a separate entrance and be kept apart from the ordinary working of the general hospital proper, so as to enable each patient who wishes to be attended in such wings by his own practitioner.

### The Union Poor-Law Hospital.

We will now turn to the consideration of another type of hospital, distinct from the Poor-Law infirmary, which is under the control of Boards of Guardians and the Local Government Board. This type is known as the Union Poor-Law hospital. Administratively it differs from the Poor-Law infirmary usually in the sense that it is not in charge of a responsible medical superintendent and his assistants, but that it is conducted largely as an extension or overflow building in connection with the workhouse, where the medical treatment of the patients is in the hands of Poor-Law medical officers, being usually general practitioners resident and practising in the district which the Union Poor-Law hospital serves. The beds in these institutions are usually largely occupied by chronic, senile, epileptic, mentally defective, and aged persons of both sexes, and here is usually found the maternity department under Poor-Law control. For the reception and care of cases of the class we have enumerated all that is required is generally reasonable accommodation, good food, and kindly treatment. The medical needs are usually of the simplest, and there is not any real demand for constant surgical treatment of the best. Hence the Union Poor-Law hospital usually has no operation theatre or casualty department, or such as it may possess is generally primitive rather than complete. We would, however, venture to call the attention of the Local Government Board, and of the Boards of Guardians immediately concerned, to the Union Poor-Law hospitals at North Evington and at Halifax and elsewhere. Taking the two institutions we have specifically mentioned, they will find, what they must already know, we imagine, that, so far as the buildings are concerned, and this is especially the case at Halifax, they represent to a large extent cleverly planned, carefully thought-out, admirably organised, and complete buildings, possessing excellent accommodation in separate buildings for the nursing staff, for the administration, for the mortuary, and for the pathological work. Nothing has been scamped in these buildings. The architect at Halifax deserves credit for having produced a really fine up-to-date hospital so far as the buildings are concerned, and if the heating apparatus of some of the wards and the operation unit were to be dealt with and brought up to date, the Halifax Union Poor-Law Hospital would be ready to enter upon a most useful career as a State hospital of the best, where acute cases of all kinds and the best surgical work could be successfully undertaken, at a moderate expenditure, with perfect efficiency.

In calling the Local Government Board's attention to these patent facts, our wish is to get them to instruct their inspectors to make a report upon every one of the present Union Poor-Law hospitals, as well as upon each Poor-Law infirmary, from the point of view of the present uses to which they are being put by (*a*) the admission of chronic, incurable, aged, and infirm people, and (*b*) for the reception and adequate treatment of acute cases of all kinds. These reports would be most valuable, because they would place the Government in possession within a few months' time of accurate data exhibiting the amount of hospital accommodation which can readily be brought into use for the purpose of treating many hundreds and probably thousands of acute cases, which the working of the Insurance Act is continuously proving require in-patient treatment. This they cannot promptly obtain in the voluntary hospitals owing to the fact that they do not contain at the present time a sufficient number of beds to meet all the demands of insured persons for in-patient treatment.

### Perpetuating the Voluntary System.

No one in Great Britain, whose opinion is entitled to carry any weight, who has studied the principles which underlie the question, who is familiar with the work or the consequences to the patients of State hospitals as they exist in other countries to-day, can fail to desire the perpetuation of the voluntary system of hospitals. To secure this it is essential to supplement the in-patient accommodation at present available by utilising in the first instance all the existing buildings which can be secured as State hospitals worked administratively on identical lines with the methods pursued in our voluntary hospitals. The first step, however, to a full realisation of the possibilities which prevail in this country in regard to in-patient hospital accommodation in existing buildings is the preparation of an accurate schedule. This schedule should contain the fullest details of the accommodation contained in great blocks of excellent buildings for the treatment of the sick, up and down the country, like those at North Evington and Halifax.

We are confident that the nation will be grateful to the President of the Local Government Board (Mr. Herbert Samuel) if he will give the necessary instructions to one or two of the most capable and knowledgeable inspectors in the Local Government Board service to proceed with this work, and render full reports to him for submission to Parliament with as little delay as possible.

